# Prediction of potential biomarkers and therapeutic targets in HIV viremic patients using immune-related circRNA-miRNA-mRNA regulatory networks

**DOI:** 10.1371/journal.pone.0355600

**Published:** 2026-08-14

**Authors:** Negin Faraji, Leila Rouhi, Azam Bolhassani, Behnam Hasannejad-asl, Kazem Baesi, Ladan Abbasian

**Affiliations:** 1 Department of Biology, ShK.C., Islamic Azad University, Shahrekord, Iran; 2 Department of Hepatitis, AIDS and Blood-borne Diseases, Pasteur Institute of Iran, Tehran, Iran; 3 Research Committee, Department of Medical Biotechnology, School of Advanced Technologies in Medicine, Shahid Beheshti University of Medical Sciences, Tehran, Iran; 4 Iranian Research Center for HIV/AIDS, Iranian Institute for Reduction of High-risk Behaviors, Department of Infectious Diseases, Imam khomeini Hospital Complex, School of Medicine, Tehran University of Medical Sciences, Tehran, Iran; Ural Federal University named after the first President of Russia B N Yeltsin Institute of Physics and Technology: Ural'skij federal'nyj universitet imeni pervogo Prezidenta Rossii B N El'cina Fiziko-tehnologiceskij institut, RUSSIAN FEDERATION

## Abstract

The recent study aims to analyze the role of non-coding RNAs, particularly circRNAs, in HIV pathogenesis using construction of immune-related regulatory networks. In compliance with ethical standards and institutional guidelines, publicly available HIV-related mRNA and microRNA expression datasets were systematically retrieved from the Gene Expression Omnibus repository. These datasets underwent rigorous bioinformatics analysis employing the “limma” package in R to identify differentially expressed genes (DEGs) and microRNAs (DEmiRNAs). Validated interaction data from miRBase and ENCORI databases facilitated the construction of circRNA-DEmiRNA and DEmiRNA-DEG regulatory networks. Protein-protein interaction networks were generated through STRING database analysis, with hub genes defined as those within the highest 5% of interaction degrees. Furthermore, single-cell RNA sequencing (scRNA-seq) datasets from HIV-infected individuals were analyzed using the “Seurat” package to identify DEGs in CD4^+^ and CD8^+^ T cell subsets. Cross-validation between microarray and scRNA-seq data ensured robustness of identified hub genes. Our results revealed substantial alterations in RNA expression associated with both high viral load and low viral load HIV infections. Single-cell analyses indicated significant gene expression shifts in T cell populations: 194 upregulated and 813 downregulated genes in CD4^+^ T cells, and 244 upregulated and 567 downregulated genes in CD8^+^ T cells. Integrated analysis delineated five hub genes consistently dysregulated across T cell subsets. STAT1 and DDX39B were upregulated, whereas CXCL8, CXCL12, and PTGS2 were downregulated. The pathway enrichment analysis indicated that these genes are implicated in key regulatory pathways relevant to infectious disease mechanisms. Alongside of DEGs, our studies indicated that multiple miRNAs, such as hsa-miR-21-5p, hsa-miR-146a-5p and hsa-let-7b-5p are differentially expressed in HIV-infected samples in comparison to the normal samples. Afterward, DEmiRNA-DEGs network studies demonstrated different axes between significantly up- and down-regulated DEGs with down- and up-regulated miRNAs, respectively. Following, ceRNA regulatory network revealed circRNAs, which can interact with DEmiRNA-DEGs networks, like hsa_circ_0089761-hsa-miR-21-5p-CXCL12, hsa_circ_0003812-hsa-miR-146a-5p-PTGS2, and has_circ_0007185–has_let-7b-5p–DDX39B. Overall, this integrative analysis suggests potential circRNA-mediated regulatory mechanisms in HIV infection and highlights potential candidate circRNAs that may serve as biomarkers or therapeutic targets for further investigation.

## Introduction

Human immunodeficiency virus (HIV) continues to be a considerable global health challenge, influencing millions of lives on a personal level. In 2024, an estimated 40.8 million people were living with HIV worldwide, according to UNAIDS (https://www.unaids.org/en, 2025). This total includes 39.4 million adults aged 15 years and older, as well as 1.4 million children aged 0–14 years (https://www.unaids.org/en, 2025). Furthermore, in 2024, 1.3 million people were newly infected with HIV. Because HIV Antiretroviral therapy (ART) successfully suppresses the spread of viruses and restores immune function, it has changed HIV-1 infection from a deadly illness to a chronic, treatable condition. However, a significant obstacle to treatment is the need for lifelong ART because persistent viral reservoirs in resting memory CD4^+^ T cells result in viral rebound when treatment is stopped [1]. New research on competing endogenous RNAs (ceRNAs), including circular RNAs (circRNAs), reveals potential regulatory roles in HIV-1 transcription and replication. Nevertheless, there are other drawbacks to circRNA research, including difficulties in detecting native circRNA forms during infection due to technological limitations, low abundance, complex splicing events, and polymerase chain reaction (PCR) bias [2]. These challenges impede a thorough comprehension of the roles of circRNA in HIV pathogenesis and treatment.

In contrast to linear RNAs, circRNAs are a class of endogenous non-coding RNAs distinguished by their covalently closed loop structures, which are created mainly by backsplicing, in which an upstream 3′ splice site connects a downstream 5′ splice site, resulting in exceptional stability. Depending on their sequence origin, circRNAs are classified into exonic circRNAs, circular intronic RNAs (ciRNAs), exon-intron circRNAs, antisense circRNAs, and mitochondrial circRNAs. Their circular structure protects them from exonuclease degradation, which contributes to their stability and integrity. Many circRNAs serve as microRNA (miRNA) sponges, binding to and sequestering miRNAs, which regulates downstream gene expression and miRNA activity. CircRNAs play a significant role in influencing cellular functions by acting as sponges for miRNAs [3–5]. MicroRNAs are a type of non-coding RNAs (ncRNA) and regulate gene expression after transcription by binding to complementary sequences on target mRNAs, resulting in either translational repression or mRNA degradation. CircRNAs act as sponges for miRNAs, sequestering them within the circRNA–miRNA–mRNA network. This process adjusts the availability of miRNAs to target mRNAs, thereby modulating gene expression. By altering protein production through miRNA-mediated silencing mechanisms, this interaction effectively regulates essential cellular functions [6–8].

Peripheral blood mononuclear cells (PBMCs), which comprise T-cells, dendritic cells (DCs), monocytes etc*.* serve as critical reservoirs for HIV. They sustain viral persistence despite ART and exhibit altered immune responses, including decreased CD4^+^ counts and dysregulated cytokine production [9]. PBMCs exhibit variable responses to HIV infection. Single-cell RNA sequencing (scRNA-seq) has demonstrated that HIV-positive patients have distinct immune cell subsets and altered gene expression profiles [10]. CircRNAs, acting as miRNA sponges, play a significant role in competing endogenous RNA (ceRNA) networks. Research on ceRNAs in PBMCs has revealed changes in miRNA and mRNA profiles associated with apoptosis and immune activation. This highlights the importance of understanding how circRNAs influences HIV pathogenesis [9]. Several circRNAs have been identified in individuals infected with HIV that act as sponges, including ciTRAN (circSMARCA5). The HIV-1 Viral Protein R (Vpr) uses ciTRAN to enhance viral replication by blocking the Serine/Arginine-rich Splicing Factor 1 (SRSF1) from the viral transcription complex, thereby preventing transcriptional repression [11]. Moreover, infected CD4^+^ T cells have been found to contain at least 15 different HIV-1-derived circRNAs [12]. These circRNAs function as molecular sponges, absorbing antiviral microRNAs—such as miR-6727-3p and miR-4722-3p—that typically aid our immune system in fighting back [12]. These results suggest that circRNAs play a crucial role in regulating HIV-1 replication and immune evasion, indicating their potential as novel therapeutic targets for HIV treatment.

**Fig 1:**
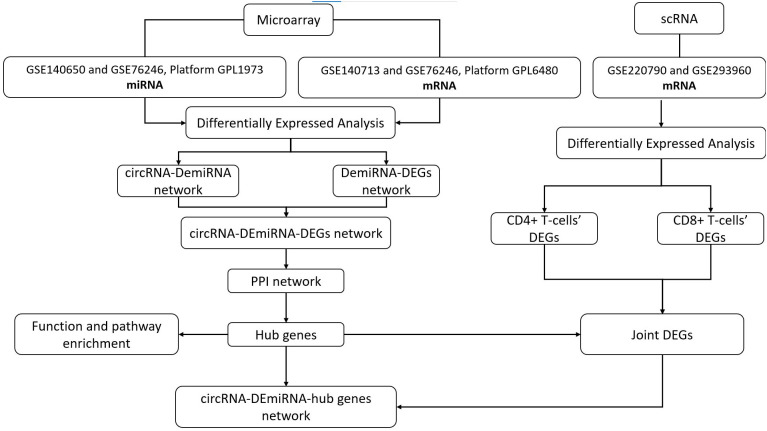
Flowchart of the steps performed in this study.

In this study, we used an integrated multi-*omics* approach to investigate the regulatory networks involved in high viral load (HVL) and low viral load (LVL) HIV-infected patients. By analyzing circulating cell microarray datasets, we systematically identified differentially expressed miRNAs (DEmiRNAs) and differentially expressed genes (DEGs) in HVL patients compared to healthy controls. These molecular profiles enabled us to construct comprehensive miRNA-mRNA networks, which we subsequently expanded to include interacting circRNAs that function as ceRNA sponges. In addition to identifying RNAs, we examined their protein-protein interactions (PPIs) to identify key hub genes, which are central players in our circRNA-miRNA-hub gene networks. Further functional enrichment analysis of these hub genes revealed interesting pathways, providing insight into how circRNAs may exert their regulatory effects. Additionally, complementary scRNA-seq analysis of immune cell populations provided cellular-resolution validation of our key findings. Fig 1 illustrates the study's flowchart. Overall, this research enhances our understanding of HIV pathogenesis by outlining novel ceRNA networks and identifying potential diagnostic biomarkers and therapeutic targets for HIV management.

## Methods

### Data acquisition

The miRNA and mRNA microarray datasets were obtained from the Gene Expression Omnibus (GEO) database. A review of the GEO and BioProject metadata was conducted to assess the provenance of the datasets and the relationships among the included studies. The dataset GSE76246 (BioProject PRJNA306750), submitted by Fudan University in Shanghai, China, contains parallel miRNA (GPL19730) and mRNA (GPL6480) expression profiles derived from PBMCs of 30 AIDS patients and 7 healthy controls. Additionally, GSE140650 (miRNA; GPL18402; BioProject PRJNA590459) and GSE140713 (mRNA; GPL6480; BioProject PRJNA590646), submitted by Sichuan University in Chengdu, China, are associated with the same originating study [13]. These datasets include PBMC samples from 16 HVL patients, 14 LVL patients, and 7 healthy controls. Detailed information regarding all datasets is provided in Table 1. Additionally, the scRNA-seq datasets GSE220790 (BioProject PRJNA911330) and GSE293960 (BioProject PRJNA1247497) were sourced from the GEO database for further analysis.

**Table 1:** Details information of datasets.

Profile	RNA type	Platform	Experimental type	Organism	Sample size			Sample type	Ref.
					HVL	LVL	Healthy		
GSE76246	mRNA	GPL6480	Expression profile by array	Homo sapiens	16	14	7	PBMCs	[13]
GSE140713	mRNA	GPL6480	Expression profile by array	Homo sapiens	16	14	7	PBMCs	
GSE76246	miRNA	GPL19730	ncRNA expression profile	Homo sapiens	16	14	7	PBMCs	
GSE140650	miRNA	GPL18402	ncRNA expression profile	Homo sapiens	16	14	7	PBMCs	

***Abbreviations:*** PBMCs, Peripheral blood mononuclear cells; HVL, High viral load; LVL, Low viral load

### Identification of differentially expressed RNA

Differentially expressed RNAs (DERNAs) were analyzed using R version 4.4 within the RStudio environment. GEO datasets were downloaded using the “GEOquery” package, and differential expression analysis was performed with the “limma” package. To assess sample clustering and identify potential outliers, principal component analysis (PCA) was performed for each dataset. After quality control, differential expression analyses were performed separately for each GEO series, using adjusted *p*-values < 0.05 and a |log2FC| ≥ 1 (indicating a ≥ 2-fold change) as significance thresholds. To identify transcriptomic features consistently detected across related public datasets, we intersected the significant results from the corresponding datasets to identify overlapping differentially expressed miRNAs and genes. Volcano plots and heatmaps were created using the “ggplot2” and “pheatmap” packages, respectively.

### Prediction of the circRNA-miRNA interaction

We obtained circRNA-miRNA interaction data from the ENCORI (The Encyclopedia of RNA Interactomes) database (https://rnasysu.com/encori/index.php) [14], which compiles experimentally validated RNA-RNA interactions from various sources, including CLIP-seq, degradome-seq, and interactome studies. This resource documents a wide range of molecular interactions, encompassing relationships such as miRNA-circRNA, miRNA-mRNA, miRNA-sncRNA, miRNA-lncRNA, and miRNA-pseudogene. Using R software, we specifically extracted the interactions involving our differentially expressed miRNAs (DEmiRNAs) for subsequent network analysis.

### Prediction of the miRNA-mRNA interaction

For the construction of the miRNA-mRNA network, we gathered interaction data from both the ENCORI database and TarBase V9.0 (https://dianalab.e-ce.uth.gr/tarbasev9) [15], which provides experimental support for miRNA targets on protein-coding transcripts. After retrieving the data, we merged the interaction datasets and removed any redundant entries to create a consolidated reference database. From this comprehensive collection, we then selected unique miRNA-mRNA interactions that involved our DEmiRNAs and DEGs for further network analysis. We utilized the “Biomart” package [16] to convert gene names from our DEGs file into Ensembl gene IDs, allowing us to match them with the database.

### Construction of the protein-protein interaction network and exploring hub genes

We obtained protein-protein interaction (PPI) data from the STRING-Version12.0 database (https://string-db.org/) [17] and analyzed it about our DEGs. Initially, we identified DEGs that interact with DEmiRNAs based on the results from above section. The resulting subset of miRNA-associated DEGs was then mapped onto the PPI network using R software. We performed network analysis with the igraph package [18] to identify hub genes based on degree centrality.

### Construction of the circRNA-miRNA-mRNA network

The circRNA-miRNA-mRNA regulatory network was constructed using the hub genes identified in above section. To create the subnetwork featuring downregulated circRNAs, upregulated miRNAs, and downregulated hub genes, we selected the downregulated hub genes and identifying their associated miRNAs from the miRNA-mRNA network discussed in above section. We then filtered these miRNAs to focus solely on the upregulated DEmiRNAs. Next, we mapped the upregulated DEmiRNAs to the circRNA-miRNA network to identify the interacting circRNAs. A similar analytical approach was employed to construct the subnetwork consisting of upregulated circRNAs, downregulated miRNAs, and upregulated hub genes. All networks were visualized using Cytoscape software (version 3.10.2) [19].

### Functional and pathway enrichment analysis of hub genes

We converted hub genes from gene symbols to ENTREZ IDs using R software. Next, we conducted a functional enrichment analysis with the “ClusterProfiler” package [20] in R, which included Gene Ontology (GO) annotations for biological processes (BP), cellular components (CC), and molecular functions (MF), as well as KEGG pathway analysis. We identified significant terms using a threshold of *p-value* < 0.05 and a minimum of three enriched genes per term. Finally, we visualized the results using the “ggplot2” package.

### Single cell exploration of DEGs in CD4^+^ and CD8^+^ T-cells

We preprocessed and analyzed the scRNA-seq data from the GSE220790 dataset (3 randomly selected patients who received ART) and the GSE293960 dataset (3 healthy samples) (Table 2) using the “Seurat” package in R to identify DEGs in CD4^+^ and CD8^+^ T-cells. The initial quality control step excluded cells based on the following criteria, including i) fewer than three detected genes, ii) mitochondrial gene content exceeding 20%, and iii) fewer than 400 detected genes per cell. The remaining cells underwent normalization, feature selection, and scaling using the SCTransform and FindVariableFeatures functions. We then performed principal component analysis using the RunPCA function, followed by batch effect correction with the Harmony package. Additionally, we conducted dimensionality reduction with RunUMAP, cell clustering using FindNeighbors and FindClusters, and removal of doublets with DoubletFinder. Cell-type annotation was performed using the HumanPrimaryCellAtlasData reference via SingleR, and differential expression analysis was conducted using FindMarkers. Herein, we utilized the same thresholds, which applied to identify the DEGs from microarray datasets (*p*-value < 0.05 and a |log2FC| ≥ 1). Finally, the results were visualized using the pheatmap and VennDiagram packages.

**Table 2:** Details information of scRNA-seq datasets.

BioProject	Profile	Platform	Samples	Status	Experimental Type	RNA type	Organism	Sample Type
PRJNA911330	GSE220790	GPL24676	GSM6817429	ART treated Patient	scRNA sequencing	mRNA	Homo Sapience	PBMC
PRJNA911330	GSE220790	GPL24676	GSM6817430	ART treated Patient	scRNA sequencing	mRNA	Homo Sapience	PBMC
PRJNA911330	GSE220790	GPL24676	GSM6817431	ART treated Patient	scRNA sequencing	mRNA	Homo Sapience	PBMC
PRJNA1247497	GSE293960	GPL24676	GSM8895287	Healthy	scRNA sequencing	mRNA	Homo Sapience	PBMC
PRJNA1247497	GSE293960	GPL24676	GSM8895289	Healthy	scRNA sequencing	mRNA	Homo Sapience	PBMC
PRJNA1247497	GSE293960	GPL24676	GSM8895292	Healthy	scRNA sequencing	mRNA	Homo Sapience	PBMC

***Abbreviations:*** PBMCs, Peripheral blood mononuclear cells

## Results

### Identification of DEmiRNAs and DEGs

**Fig 2:**
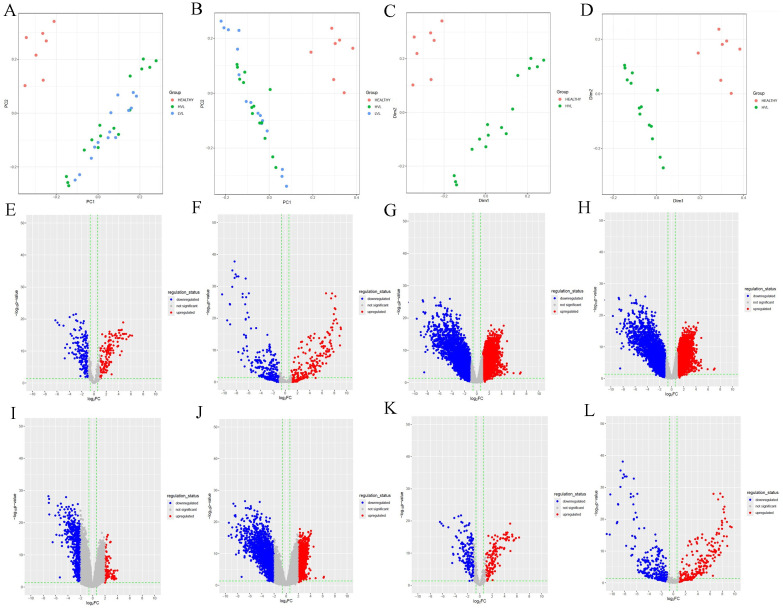
The principal component analysis and volcano plot of the DEmiRNA and DEGs between HVL- and LVL-HIV conditions and healthy samples. (A) The principal component analysis of GSE76246-GPL19730; (B) The principal component analysis of GSE18402; (C) The principal component analysis of GSE76246-GPL6480; (D) The principal component analysis of GSE140713; (**E)** volcano plot of DEmiRNA from GSE76246-GPL19730 in HVL-HIV-related condition; (**F)** volcano plot of DEmiRNA from GSE18402 in HVL-HIV-related condition; (**G)** volcano plot of DEGs from GSE76246-GPL6480 in HVL-HIV-related condition; (**H)** volcano plot of DEGs from GSE140713 in HVL-HIV-related condition; (**I)** volcano plot of DEGs from GSE76246-GPL6480 in LVL-HIV-related condition; (**J)** volcano plot of DEGs from GSE140713 in LVL-HIV-related condition; (**K)** volcano plot of DEmiRNA from GSE76246-GPL19730 in LVL-HIV-related condition; (**L)** volcano plot of DEmiRNA from GSE18402 in LVL-HIV-related condition. *Abbreviations:* DEmiRNA: Differentially Expressed miRNA; **DEGs**: Differentially Expressed Genes; HVL: High Viral Load; LVL: Low Viral Load.

In the present study, we conducted a comprehensive reanalysis of publicly available HIV-related transcriptomic datasets to identify DEmiRNAs and DEGs associated with both HVL and LVL status. Differential expression analysis was performed individually for each GEO series, following quality control procedures that included principal component analysis (PCA). The PCA revealed a clear distinction between HIV-positive samples and healthy control samples across the datasets (Fig 2A–D). To identify robust transcriptomic features, we determined the overlapping differentially expressed RNAs across the related GEO datasets. For the HVL samples, we identified 271 overlapping DEmiRNAs, including 136 upregulated and 135 downregulated (Supplementary S1 File). Additionally, we identified 3,208 overlapping DEGs, comprising 1,571 upregulated and 1,637 downregulated genes relative to healthy controls (Supplementary S1 File). The corresponding volcano plots are shown in Fig 2E–H. For the LVL samples, we identified 278 overlapping DEmiRNAs, including 128 upregulated and 150 downregulated (Supplementary S2 File). We also identified 3,267 overlapping DEGs, comprising 1,620 upregulated genes and 1,647 downregulated genes compared with healthy controls (Supplementary S2 File). The corresponding volcano plots are presented in Fig 2I–L.

### Prediction of circRNA-miRNA and miRNA-mRNA interactions

We utilized the ENCORI and TarBase databases as reliable sources of molecular interaction data to create circRNA-miRNA and miRNA-mRNA regulatory networks for the DEmiRNAs and DEGs identified in HIV-infected samples (*i.e.,* HVL and LVL). In our analysis of the circRNA-miRNA network under the HVL condition, we found that 143 unique DEmiRNAs interacted with multiple circRNAs. Further examination of the miRNA-mRNA interaction network showed that 97 of these 143 DEmiRNAs were associated with 2,423 DEGs (Supplementary S3 File). In contrast, the circRNA-miRNA network in the LVL condition included 104 unique DEmiRNAs, with 101 of these interacting with 1,149 DEGs in the miRNA-mRNA network (Supplementary S4 File).

### Construction of the protein-protein interaction network and definition of hub genes

The PPI networks were constructed using DEGs derived from the DEmiRNA-DEG regulatory networks for both HVL and LVL conditions of HIV infection. PPI data were retrieved from the STRING database, and mapped to filtered DEGs using R software. In the HVL-HIV study, after excluding non-interacting genes, the PPI network comprised 2,516 nodes and 295,629 edges, representing interactions among 2,383 of the 2,423 input DEGs. After filtering-out the housekeeping genes based on Housekeeping genes and Reference Transcripts Atlas (HRT), the network topology analysis conducted with the “igraph” package in R identified the top 5% of hub genes based on degree centrality, resulting in 102 hub genes, 67 upregulated and 35 downregulated. The some top up- and down-regulated hub genes by connectivity are Axis Inhibition Protein 1 (AXIN1), Caseinolytic Mitochondrial Matrix Peptidase Chaperone Subunit B (CLBP), Ras Homolog Family Member U (RHOU), RNA polymerase III subunit B (POLR3B), and Interferon regulatory factor 1 (IRF1). Detailed information on the top ten-upregulated and downregulated hub genes in HVL samples is provided in Table 3. In contrast, the LVL-HIV PPI network included 1,156 nodes and 64,866 edges, capturing interactions among 1,121 of the 1,149 DEGs analyzed. The top 5% of hub genes identified in this network comprised 18 upregulated and 35 downregulated genes, with Flap structure-specific endonuclease 1 (FEN1), Integrin subunit beta 2 (ITGB2), Cytochrome b-245 beta chain (CYBB), POLR3B, and IRF1 being the highest connectivity nodes. Comprehensive data for the top ten-upregulated and downregulated hub genes in LVL samples are summarized in Table 3.

**Table 3:** Information of top10 upregulated and downregulated hub genes in HIV-infected patients.

HVL-HIV			
Upregulated	Degree	Downregulated	Degree
E1A binding protein p300 (EP300)	1073	RNA polymerase III subunit B (POLR3B)	700
CD8 alpha chain (CD8A)	1020	Interferon regulatory factor 1 (IRF1)	697
MDM2 proto-oncogene (MDM2)	1008	Isocitrate dehydrogenase (NADP (+)) 1 (IDH1)	695
Ribonuclease L (RNASEL)	1006	CCAAT/enhancer binding protein alpha (CEBPA)	693
Signal transducer and activator of transcription 1 (STAT1)	941	Filamin A (FLNA)	688
Rac family small GTPase 2 (RAC2)	938	Caspase 8 (CASP8)	684
Mitogen-activated protein kinase 14 (MAPK14)	930	Histone deacetylase 4 (HDAC4)	676
Cyclin B1 (CCNB1)	894	Bruton tyrosine kinase (BTK)	674
Cyclin G associated kinase (GAK)	884	RuvB like AAA ATPase 2 (RUVBL2)	666
Transforming growth factor beta receptor 2 (TGFBR2)	838	CD68 molecule (CD68)	654
**LVL-HIV**			
Flap structure-specific endonuclease 1 (FEN1)	620	RNA polymerase III subunit B (POLR3B)	700
Integrin subunit beta 2 (ITGB2)	612	Interferon regulatory factor 1 (IRF1)	697
Cytochrome b-245 beta chain (CYBB)	604	Filamin A (FLNA)	688
RB binding protein 4 (RBBP4)	602	CCAAT/enhancer binding protein alpha (CEBPA)	693
Cell division cycle associated 8 (CDCA8)	602	Caspase 8 (CASP8)	684
DExD-box helicase 39B (DDX39B)	596	Bruton tyrosine kinase (BTK)	674
Thymidylate synthetase (TYMS)	594	Histone deacetylase 4 (HDAC4)	676
NOP56 ribonucleoprotein (NOP56)	590	RuvB like AAA ATPase 2 (RUVBL2)	666
IKAROS family zinc finger 1 (IKZF1)	587	CD68 molecule (CD68)	654
E1A binding protein p400 (EP400)	589	Tyrosine kinase 2 (TYK2)	644

### CircRNA-miRNA-mRNA network construction

**Fig 3:**
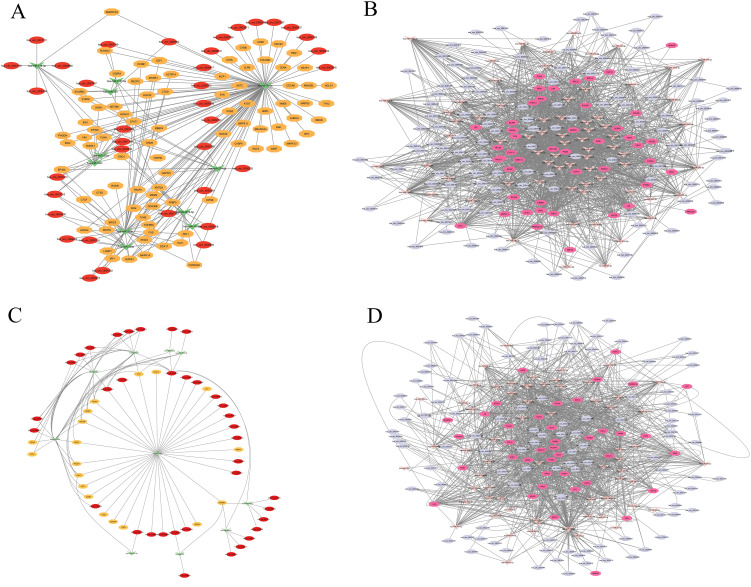
HIV-associated circRNA-miRNA-mRNA Regulatory Networks. (**A**) circRNA-downmiR-up hub genes in HVL-HIV-related condition; (**B**) circRNA-upmiR-down hub genes in HVL-HIV-related condition; (**C**) circRNA-downmiR-up hub genes in LVL-HIV-related condition; (**D**) circRNA-upmiR-down hub genes in LVL-HIV-related condition. In all panels, hub genes are represented as octagons. In panels A and C, upregulated hub genes are shown in orange, while downregulated hub genes in panels B and D are displayed in mauve. CircRNAs are illustrated as ovals, with upregulated circRNAs in red for panels A and C, and downregulated ones in purple for panels B and D. miRNAs are depicted in a V shape; they are colored green for upregulated miRNAs in panels A and B, and light pink for downregulated ones in panels C and D. *Abbreviations:* HVL: High Viral Load; LVL: Low Viral Load.

To clarify the molecular mechanisms by which circRNAs contribute to the progression of HIV infection, we constructed ceRNA networks for both upregulated and downregulated hub genes identified in studies with HVL and LVL HIV infections. In the HVL-HIV studies, we established two distinct ceRNA networks. The first network includes circRNAs, downregulated miRNAs, and upregulated hub genes, comprising 11 downregulated miRNAs, 86 upregulated hub genes, and 36 circRNAs (Fig 3A). The second network features circRNAs, upregulated miRNAs, and downregulated hub genes, consisting of 72 upregulated miRNAs, 46 downregulated hub genes, and 121 circRNAs (Fig 3B). For the LVL-HIV condition, the first ceRNA network integrates circRNAs, downregulated miRNAs, and upregulated hub genes, which include 10 downregulated miRNAs, 19 upregulated hub genes, and 36 circRNAs (Fig 3C). The second LVL network comprises circRNAs, upregulated miRNAs, and downregulated hub genes, involving 66 upregulated miRNAs, 40 downregulated hub genes, and 114 circRNAs (Fig 3D). These ceRNA networks may provide a preliminary framework for understanding the potential regulatory roles of circRNAs in HIV infection and offer insights into biological pathways that could be involved in disease progression, although further experimental validation is required.

### Functional and pathway enrichment analysis of hub genes

**Fig 4:**
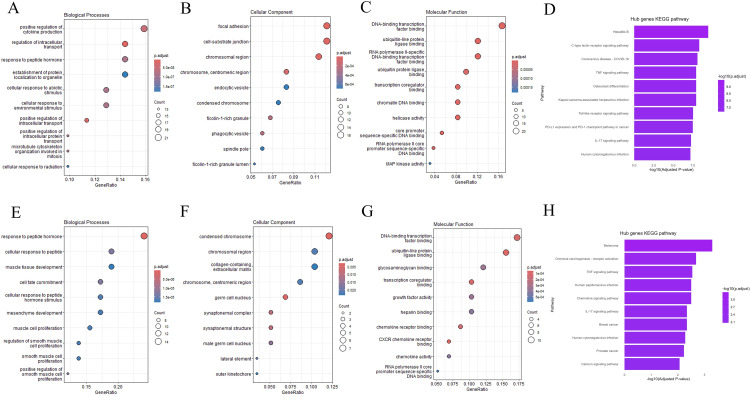
Functional and pathway enrichment analysis of hub genes that identified in the PPI network. (A) GO enrichment analysis of biological processes highlights immune-related pathways in HVL-HIV-related condition; (B) GO analysis of cellular components emphasizes key subcellular localizations in HVL-HIV-related condition; (C) GO analysis of molecular functions reveals critical binding activities; in HVL-HIV-related condition (D) KEGG pathway enrichment analysis illustrates significantly altered signaling pathways in HIV infection in HVL-HIV-related condition; (E) GO enrichment analysis of biological processes highlights immune-related pathways in LVL-HIV-related condition; (F) GO analysis of cellular components emphasizes key subcellular localizations in LVL-HIV-related condition; (G) GO analysis of molecular functions reveals critical binding activities; in LVL-HIV-related condition (H) KEGG pathway enrichment analysis illustrates significantly altered signaling pathways in HIV infection in LVL-HIV-related condition. The bubble **plots (A-C and E-G)** display enrichment significance (*adjusted p-values*) versus gene ratio, with the size of each bubble representing the number of genes. The bar plot **(D and H)** visualizes the relationships between pathways and genes, where the height of the bars corresponds to enrichment significance, and the color gradient indicates the adjusted -log10 (*p.adjust*). *Abbreviations:* PPI: protein-protein interaction; GO: Gene Ontology; HVL: High Viral Load; LVL: Low Viral Load.

To elucidate the biological functions of hub genes identified in HVL and LVL HIV conditions, Gene Ontology (GO) annotation and KEGG pathway enrichment analyses were conducted utilizing the “ClusterProfiler” R package. In the HVL-HIV cases, GO enrichment highlighted significant associations within three principal categories, including: a) Biological processes (BP), including positive regulation of cytokine production, regulation of intracellular transport, and response to peptide hormone; b) Cellular components (CC), such as focal adhesion, cell-substrate junction, and chromosomal region; and c) Molecular functions (MF), notably DNA-binding transcription factor binding, ubiquitin-like protein ligase binding, and RNA polymerase II-specific DNA-binding transcription factor binding. Complementary KEGG pathway analysis identified key pathways involving Hepatitis B infection, C-type lectin receptor signaling pathway, and Coronavirus disease-COVID-19 (Fig 4A-D). Conversely, in the LVL-HIV group, GO terms significantly enriched encompassed, containing i) Biological processes including response to peptide hormone, cellular response to peptide, and muscle tissue development; ii) Cellular components such as condensed chromosome, chromosomal region, and collagen-containing extracellular matrix; and iii) Molecular functions, especially DNA-binding transcription factor binding, ubiquitin-like protein ligase binding, and glycosaminoglycan binding. Corresponding KEGG analysis revealed crucial pathways related to Melanoma, chemical carcinogenesis, receptor activation, and TNF signaling pathway (Fig 4E-H).

### ScRNA-seq analysis

**Fig 5:**
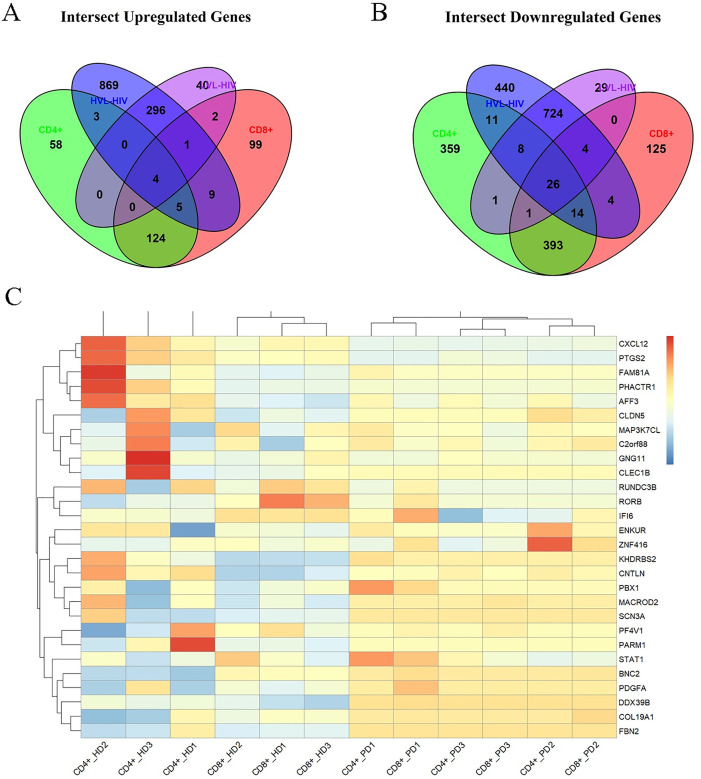
Multi-platform transcriptomic profiling reveals signatures associated with HIV. (A) Venn diagram showing the integrated upregulated genes across HVL- and LVL-HIV related conditions, CD4^+^ and CD8^+^ T-cells; (B) Venn diagram showing the integrated downregulated genes across HVL- and LVL-HIV related conditions, CD4^+^ and CD8^+^ T-cells; (C) Heatmap depicting the integrated DEGs in the microarray and scRNA-seq studied cells. The color gradients in the heatmap indicate relative expression levels (red: high; blue: low), while the branches of the dendrogram demonstrate transcriptional similarities between various cell states. *Abbreviations:* DEGs: differentially expressed genes; scRNA-seq: single-cell RNA sequencing; HVL: High Viral Load; LVL: Low Viral Load.

We conducted scRNA-seq analysis on PBMCs obtained from HIV-infected and healthy donors to investigate CD4^+^ and CD8^+^ T cells. After applying rigorous quality control, data normalization, and batch effect correction using the Harmony algorithm (S1-S3 Figs), we observed significant differential gene expression patterns between the HIV-infected and control samples. Specifically, HIV-infected CD4^+^ T cells exhibited 194 genes that were significantly upregulated and 813 genes that were downregulated when compared to healthy controls. In contrast, CD8^+^ T cells showed 244 upregulated genes and 567 downregulated genes relative to the same healthy controls. Among these DEGs, 61 were uniquely upregulated in CD4^+^ T cells, while 111 were uniquely upregulated in CD8^+^ T cells. Additionally, 379 genes were uniquely downregulated in CD4^+^ T cells, and 133 were uniquely downregulated in CD8^+^ T cells. Moreover, we identified a subset of 128 genes that showed concordant upregulation and 434 genes that displayed concordant downregulation across both T-cell populations (Supplementary S5 File). Exploring the joint upregulated and downregulated DEGs of CD4^+^ and CD8^+^ T-cells with HVL- and LVL-HIV conditions resulted in the 4 upregulated and 26 downregulated genes (Figs 5a and 5b). The expression patterns of these genes are illustrated in the heatmap shown in Fig 5c.

### Differentially expressed hub genes in CD4^+^ and CD8^+^ T-cells

**Fig 6:**
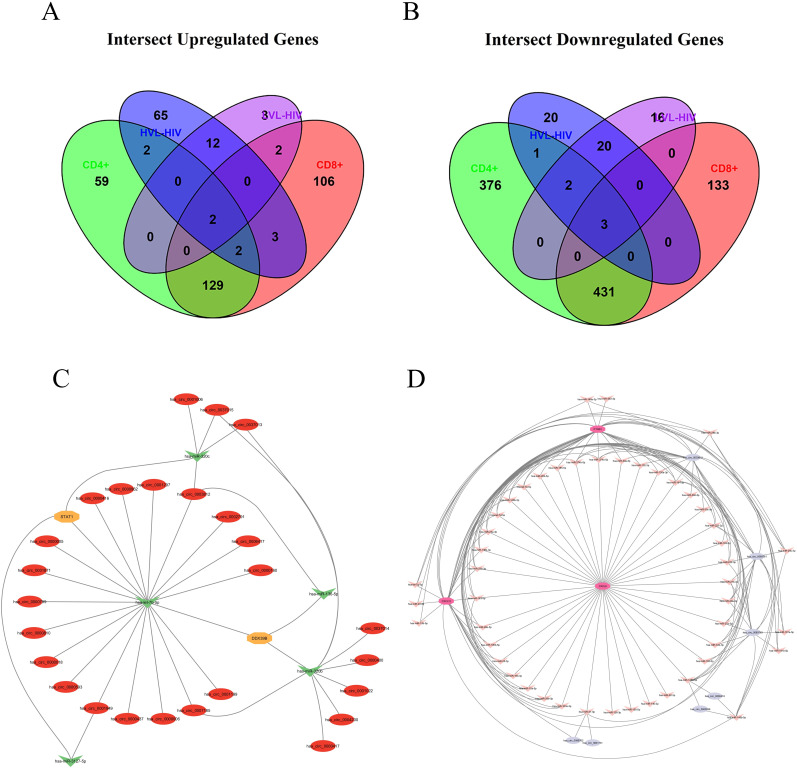
Multi-platform transcriptomic profiling reveals signatures associated with HIV. (A) Venn diagram showing the integrated upregulated hub genes across HVL- and LVL-HIV related conditions, upregulated genes across CD4^+^ and CD8^+^ T-cells; (B) Venn diagram showing the integrated downregulated hub genes across HVL- and LVL-HIV related conditions, downregulated genes across CD4^+^ and CD8^+^ T-cells; (C) HVL- and LVL-HIV, CD4^+^ and CD8^+^ T-cells integrated circRNA-downmiR-up hub genes; (D) HVL- and LVL-HIV, CD4^+^ and CD8^+^ T-cells integrated circRNA-upmiR-down hub genes; In panels C upregulated hub genes are shown in orange, while downregulated hub genes in panels D displayed in mauve. CircRNAs are illustrated as ovals, with upregulated circRNAs in red for panel C, and downregulated ones in purple for panel D. miRNAs are depicted in a V shape; they are colored green for upregulated miRNAs in panel C, and light pink for downregulated ones in panel D. *Abbreviations:* HVL: High Viral Load; LVL: Low Viral Load.

We aimed to integrate microarray analysis with scRNA-seq data to identify key hub genes within PBMCs associated explicitly with CD4^+^ and CD8^+^ T-cells. Our comparative analysis revealed 14 hub genes that were consistently upregulated in both HVL and LVL HIV conditions. Notably, DDX39B and STAT1, which are key regulators of antiviral immune responses, showed significant upregulation in both CD4^+^ and CD8^+^ T-cell populations (Fig 6a). In contrast, we found 25 hub genes that were consistently downregulated in both HVL and LVL contexts. Among these, CXCL8 and CXCL12, which encode essential chemokines, as well as PTGS2, both of which are critically involved in immune modulation and inflammatory processes, exhibited significant downregulation in both T-cell subsets (Fig 6b). Furthermore, based on circRNA-DEmiRNA-DEGs regulary networks in above section, the identified DEGs interacted with various DEmiRNAs, such as hsa-miR-21-5p, hsa-miR-146a, and hsa-let-7b-5p. These miRNAs are recognized as pivotal players in the pathogenesis of infectious diseases like HIV (Figs 6c and 6d). Overall, our integrative analyses outline a robust network of T-cell-associated hub genes and miRNAs that may inform future targeted therapeutic strategies while adhering to data integrity and reproducibility standards required by scientific publishing ethics.

## Discussion

The ongoing global challenge of HIV infection necessitates innovative diagnostic biomarkers and therapeutic strategies. However, recent research has highlighted ncRNAs as promising diagnostic biomarkers and potential therapeutic targets in HIV pathogenesis. Advances in RNA sequencing technologies have demonstrated that circRNAs play a significant role in critical regulatory networks during HIV infection [21]. However, the specific mechanistic roles of circRNAs in HIV-related immune dysregulation are not fully understood, yet indicating a significant gap in our knowledge of viral pathogenesis.

The objective of this study was to systematically analyze publicly available microarray expression datasets to identify DEGs and DEmiRNAs in patients with HVL and LVL states. In addition, scRNA-seq datasets were analyzed to characterize DEGs in key immune cell subsets, including CD4+ and CD8 + T lymphocytes, and to integrate these findings with the microarray-derived results. Based on the identified DEmiRNAs, circRNA–miRNA interactions were retrieved from the ENCORI database to infer potential circRNA-associated regulatory relationships. These data were subsequently used to construct a putative circRNA–miRNA–mRNA network, intended to generate hypotheses regarding regulatory mechanisms that may be involved in host responses to different viremic states.

In this study, through integrated bioinformatics analysis of PBMC samples from individuals with HVL and LVL of HIV, we identified 271 DEmiRNAs and 3,208 DEGs in HVL-HIV samples, and 278 DEmiRNA and 3,267 DEGs in LVL-HIV samples. The analysis of PPIs among DEGs revealed 132 significant hub genes for HVL-HIV condition. These genes were integrated into a comprehensive regulatory network that included 130 circRNAs, 83 DEmiRNAs, and 132 hub genes. In contrast, the PPI analysis related to LVL-HIV identified 59 significant hub genes, which were incorporated into a similar regulatory framework that comprised 125 circRNAs, 76 DEmiRNAs, and 59 hub genes. The overall architecture of the network demonstrates extensive connectivity between non-coding RNAs and coding RNAs, showcasing distinct subnetwork configurations for both upregulated and downregulated elements. These findings highlight the complex post-transcriptional regulatory mechanisms involved in HIV pathogenesis and provide a solid foundation for further exploration of molecular interactions in this context.

Additionally, we analyzed mRNA expression profiles in CD4^+^ and CD8^+^ T-cells, which play essential roles in the body's defense against viral infections. Our findings revealed that several genes, such as JAK2, VNN1, and S100A12, were significantly downregulated in both CD4^+^ and CD8^+^ T-cells. Additionally, some genes, such as SOCS3, were upregulated in both cell types, indicating shared regulatory patterns. These genes exhibit critical roles in viral infections, particularly HIV, influencing pathogenesis, immune evasion, and therapeutic potential. For example, JAK2 encodes a tyrosine kinase essential for the JAK-STAT signaling pathway, which regulates CD4^+^ and CD8^+^ T-cell activation, proliferation, and cytokine responses during viral infections, including HIV. Several studies show that JAK2 modulates immune defense by mediating cytokine receptor signaling, influencing antiviral responses and inflammation. Dysregulation of JAK2 can impair T-cell function, potentially affecting HIV pathogenesis and immune control [22–24]. While, S100A12, a calcium-binding protein, chiefly produced by neutrophils and monocytes, plays a significant role in immune response regulation, it activates inflammatory pathways through binding to receptors like RAGE and TLR2, leading to cytokine production and immune cell recruitment [25,26]. High S100A12 expression correlates with increased inflammation and altered T-cell activity, potentially impacting CD4^+^ and CD8^+^ T-cell responses in viral infections, including HIV, by modulating immune activation and inflammatory status [27]. Also, VNN1 (Vanin-1) is involved in regulating inflammation and oxidative stress, which affects immune activation and the progression of diseases caused by viral infections, including HIV [28,29]. The SOCS3 gene encodes a protein that suppresses cytokine signaling, playing a crucial role in regulating immune responses. Some studies showed that SOCS3 is upregulated during various viral infections, including HIV, where it inhibits the JAK-STAT pathway and interferon signaling. This suppression can help the virus evade the immune response, impairing antiviral defenses and potentially promoting HIV replication. Therefore, SOCS3 serves as a key negative regulator of antiviral immunity, and its dysregulation may contribute to the pathogenesis of HIV [30–32]. Among the CD4^+^ and CD8^+^ T-cells shared DEGs, some of them, like CXCL12, PTGS2, and DDX39, were identified as hub genes in our HVL- and LVL-HIV microarray studies.

Following differential expression analysis of miRNA and gene profiles, we constructed regulatory circRNA–DEmiRNA–hub gene networks. Integrative analysis across HVL and LVL HIV conditions, and CD4^+^ and CD8^+^ T cell subsets revealed consistent downregulation of CXCL12 and PTGS2 across all examined platforms. In our network analysis involving upregulated miRNA and downregulated hub genes, we identified a significant interaction between hsa_circ_0089761, hsa-miR-21-5p, and CXCL12, which may play a crucial role in infection conditions. The hsa-miR-21-5p is known to significantly influence the progression of HIV infection by modulating pathways related to inflammation and oxidative stress. Several studies have shown that the levels of miR-21-5p are elevated in the serum of HIV-infected individuals, including treatment-naive patients and even elite controllers [33]. This suggests the miR-21–5 p's continued involvement in HIV pathogenesis despite ART. Researchers utilizing next-generation sequencing (NGS) have identified elevated levels of circulating miR-21-5p in HIV patients. This increase is associated with chronic inflammation and immune dysregulation, which are significant contributors to HIV-related comorbidities [33]. Mechanistically, miR-21-5p targets several pathways, including MAPK signaling, cytokine-cytokine receptor interactions, and the HIV-1 Nef-mediated modulation of apoptosis, thereby influencing immune activation and viral persistence [33]. Additionally, another study pointed out that the altered expression of miR-21-5p correlates with complications in bone metabolism among HIV-positive individuals [34]. Overall, these findings highlight miR-21-5p as a crucial regulator of HIV-induced inflammation and indicate its potential as a biomarker or therapeutic target for managing the progression of HIV. CXCL12 that identified as a target of miR-21-5p, in this study, plays a crucial role in HIV infection by binding to the chemokine receptor CXCR4. This binding prevents HIV-1 entry into CD4^+^ T lymphocytes, particularly for CXCR4^-^tropic (X4) strains, by inhibiting the viral gp120 from interacting with CXCR4 [35]. As a result, this inhibition reduces viral fusion and disrupts transcription during the early stages of infection. However, HIV-1 variants that are resistant to CXCL12 can emerge, especially in the later stages of the disease. These resistant variants enable enhanced entry into naive CD4^+^ T cells despite the presence of CXCL12, which contributes to the depletion of CD4^+^ T cells and progresses the disease [35]. These resistant viruses exploit CXCR4 conformations that are less accessible to CXCL12, leading to immune dysregulation [36]. Additionally, CXCL12 regulates immune cell migration and inflammation through CXCR4 signaling, further influencing HIV pathogenesis and neuroinflammation [36]. These studies highlight CXCL12's dual role in both restricting HIV spread and enabling viral evasion, emphasizing its potential as a therapeutic target. Recent studies have underscored the critical role of circRNAs in the progression of viral infections, particularly HIV. Network studies have shown that hsa_circ_0089761 directly interacts with miR-21-5p. This specific circRNA, encoded by mitochondrial DNA, plays a crucial role in regulating immune responses during infections by modulating inflammatory signaling pathways. Evidence suggests that hsa_circ_0089761 is downregulated in hepatic stellate cells when stimulated by lipopolysaccharides (LPS), implying a role in controlling inflammation in infectious contexts [37]. Although direct evidence regarding the role of hsa_circ_0089761 in specific infectious diseases remains limited, its predicted regulatory associations with immune and fibrotic pathways [37] suggest a possible involvement in tissue responses to infection; however, this interpretation should be considered preliminary and requires experimental validation.

Another RNA interaction linked to circRNA-up_miR_down_hub_genes network is hsa_circ_0003812-hsa-miR-146a-5p-PTGS2. It was showed that hsa-miR-146a-5p is upregulated in HIV-infected macrophages and contributes to disease progression by targeting CCL5, a chemokine crucial for monocyte migration [38]. This targeting reduces the recruitment of immune cells and facilitates viral persistence. Additionally, miR-146a-5p was shown to suppress the production of antiviral cytokines, such as IFN-γ, IL-2, and TNF-α, as well as cytotoxicity in CD8^+^ T cells, promoting immune exhaustion in chronic HIV infection. Blocking miR-146a can restore these immune functions *ex vivo*, highlighting its role in immune suppression [39]. Another study confirmed the involvement of miR-146a in immune regulation during HIV infection, emphasizing its potential as a biomarker for immune exhaustion and a therapeutic target to enhance antiviral immunity [39,40]. These findings reveal miR-146a-5p as a key modulator of HIV pathogenesis, influencing immune suppression and altered cell migration. Our analysis revealed that miR-146a interacts with PTGS2, a key player in HIV infection. Recent studies emphasize the critical role of PTGS2 (also known as COX-2) in the development of viral infections. Its downregulation significantly influences disease progression and immune responses. In the context of HIV, transcriptomic profiling of naïve CD4^+^ T cells from both early and late HIV progressors showed a notable downregulation of PTGS2 in early progressors. This suggests a link between PTGS2 levels and altered immune regulation during the infection [41]. Additionally, broader analyses of other viral infections, such as SARS-CoV-2, reveal that PTGS2 expression is intricately regulated. Dysregulation of PTGS2 contributes to inflammatory responses and disease severity [42]. These findings highlight the importance of PTGS2 as a molecular marker and a potential therapeutic target in viral diseases, particularly HIV. Here, we showed hsa_circ_0003812 to interact directly with miR-146a. Although there is limited research specifically studying hsa_circ_0003812 in the context of infections, related circular RNAs, such as hsa_circ_0004812, have been found to modulate cytokine storms in COVID-19 by sponging miRNAs and affecting immune signaling pathways [43]. This suggests that hsa_circ_0003812 may also influence host-pathogen interactions and immune regulation during infections, and further research is necessary to clarify its specific mechanisms and roles in infectious diseases like HIV.

In addition to identifying intersecting downregulated hub genes, we observed that DDX39B was consistently upregulated across all analyzed platforms. Consequently, we examined the regulatory network comprising circRNAs, downregulated microRNAs, and DDX39B, resulting in the identification of the has_circ_0007185–has_let-7b-5p–DDX39B axis. The hsa-let-7b-5p plays a significant regulatory role in viral infections, particularly in the progression of HIV. Early studies indicated that HIV infection downregulates let-7 family miRNAs in CD4^+^ T cells, leading to increased IL-10 expression and immune dysregulation, which in turn facilitates viral persistence and immune escape [44,45]. These findings emphasize let-7b-5p as a critical modulator of HIV pathogenesis and suggest it could be a potential therapeutic target. DDX39, particularly its paralog DDX39A (also known as URH49), is a direct target of let-7b-5p and plays a complex role in HIV infection and progression. DDX39A is involved in pre-mRNA splicing and mRNA export as part of the AREX complex. It acts as a negative regulator of innate immune signaling by binding to antiviral transcripts and sequestering them in the nucleus, thereby limiting their transcription and subsequent expression [46,47]. During HIV-1 infection, DDX39A expression is increased in primary CD4^+^ T cells, suggesting it plays a role in modulating the host's antiviral responses. Additionally, siRNA screens have identified DDX39A as a host factor that can inhibit HIV-1 replication. Its upregulation during viral latency indicates it contributes to maintaining viral persistence [48]. Meanwhile, its paralog, DDX39B, associates with unspliced HIV-1 RNA but is inhibited by the viral protein Rev. This inhibition prevents the recruitment of the TREX complex, which impacts viral RNA trafficking [48]. These findings highlight that DDX39 proteins are crucial regulators of HIV RNA metabolism and immune evasion, rendering them as potential therapeutic targets. Based on the presented interaction, hsa_circ_0007185 interacts with let-7b-5p. Hsa_circ_0007185 is a regulatory circRNA that may play a role in immune responses and inflammation. Given that direct studies on hsa_circ_0007185 are limited, its potential role as a diagnostic biomarker or therapeutic target in infectious diseases remains speculative and warrants further experimental investigation.

In addition to our network studies, we examined the pathway enrichment of hub genes. Our HVL-HIV condition analysis revealed that hub genes such as STAT3 and AKT2 are linked to pathways, including “Hepatitis B,” “Toll-like receptor signaling” and “TNF signaling.” These hub genes play critical roles in their respective pathways, as evidenced by their functional mechanisms. STAT3 activated downstream of TNFR1, modulates cytokine production by inducing the anti-inflammatory mediator TNFAIP3/A20. This process helps balance pro- and anti-inflammatory responses in TNF signaling, contributing to immune regulation during chronic inflammation and cancer [49–51]. In addition, AKT2 is involved in HBV pathogenesis via the PI3K-Akt pathway, which promotes cell survival, supports viral replication, and participates in TLR-mediated metabolic reprogramming of immune cells, thus enhancing antiviral responses [52,53]. LVL-HIV-related hub genes, such as CXCL8 and STAT1, are associated with pathways including Human Papillomavirus Infection, Chemokine Signaling Pathways, and Human Cytomegalovirus Infection. During HIV infection, CXCL8 enhances viral replication in macrophages and microglia by activating its receptors, CXCR1 and CXCR2, which facilitates effective viral infection [54]. Similarly, STAT1 plays a crucial role by activating the transcription of numerous antiviral genes and enhancing immune functions, such as the upregulation of T-bet and the production of memory B cells [55–57]. Many viruses have evolved strategies to inhibit STAT1 to evade host immunity, highlighting its significance in antiviral responses [57].

Overall, this study explores the regulatory mechanisms of circRNA-mediated ceRNA networks in the context of HIV pathogenesis and their relationship with immune cell infiltration patterns. Through extensive bioinformatics analysis, we have developed a framework for understanding how ceRNA networks contribute to immune dysregulation caused by HIV. Our computational approach successfully identified differentially expressed RNAs and reconstructed their interaction networks. However, further experimental validation is essential. Key mechanistic studies should include quantitative reverse transcription polymerase chain reaction (qRT-PCR) to verify RNA expression, western blotting for protein-level confirmation, dual-luciferase reporter assays for direct target validation, and functional experiments involving the overexpression or knockdown of network components. These steps will be crucial in substantiating the biological relevance of the identified ceRNA axes.

In conclusion, this study analyzed the expression profiles of HVL and LVL HIV conditions and integrated the results with the expression profiles of CD4^+^ and CD8^+^ T cells. Our results revealed substantial alterations in RNA expression associated with both high viral load and low viral load HIV infections. For HVL samples, 271 DEmiRNAs and 3,208 DEGs were identified, leading to the construction of multi-tiered regulatory networks, including a circRNA-DEmiRNA network with 99 circRNAs and 143 DEmiRNAs, a DEmiRNA-DEG network including 97 DEmiRNAs and 2,423 DEGs, and a PPI network featuring 132 hub genes. Integration generated a circRNA-DEmiRNA-hub gene network encompassing 130 circRNAs, 83 DEmiRNAs, and 132 hub genes. In LVL samples, parallel networks were established involving 148 circRNAs, 104 DEmiRNAs, and 1,149 DEGs, with 59 hub genes identified within the PPI framework. Corresponding ceRNA networks included 125 circRNAs, 76 DEmiRNAs, and 59 hub genes. As a result, we constructed circRNA-DEmiRNA-hub gene regulatory networks to clarify the molecular mechanisms of HIV infection. Our integrated analysis identified several functionally relevant circRNA-miRNA-mRNA regulatory networks in HIV infection, including the hsa_circ_0089761-hsa-miR-21-5p-CXCL12, hsa_circ_0003812-hsa-miR-146a-5p-SOCS3, and hsa_circ_0007185-hsa-let-7b-5p-DDX39. These identified ceRNA networks showed significant positive correlations with selected immune cell populations, suggesting a potential association with HIV-related immune modulation. Overall, this work may contribute to a preliminary understanding of the molecular mechanisms potentially involved in HIV pathogenesis and may help prioritize candidate biomarkers and therapeutic targets for further investigation. While the results highlight possible avenues for future research, they do not establish causal relationships or clinical applicability at this stage. The analyses were conducted in accordance with relevant ethical and institutional data use guidelines. The findings presented here provide a basis for subsequent experimental validation studies, which are necessary to confirm the biological and clinical relevance of the identified regulatory networks.

### Limitations

This study is primarily based on the integrative analysis of publicly available transcriptomic datasets, which introduces several inherent limitations. Variability across datasets, including differences in sample size, patient heterogeneity, experimental platforms, and data processing methods, may influence the identification of DEGs and DEmiRNAs. In addition, because the analysis integrated datasets generated from different studies and platforms, dataset provenance and sample annotations were carefully reviewed to minimize the risk of inappropriate reuse of the same biological samples across the mRNA and miRNA analyses; however, any residual cohort overlap or unrecognized differences in cohort composition may still affect the interpretation of the results. Moreover, the construction of circRNA–miRNA–mRNA regulatory networks relies on established databases and *in silico* prediction tools, which may include false-positive or incomplete interaction information and therefore should be interpreted with caution. In addition, the absence of independent cohort validation may affect the generalizability of the findings. Finally, the results of this study have not yet been experimentally validated, and further mechanistic investigations are required to confirm the biological and clinical relevance of the identified regulatory networks.

## Supporting information

S1 File Intersect differential expression analysis in HVL samples relative to healthy controls. (XLSX)

S2 File Intersect differential expression analysis in LVL samples relative to healthy controls. (XLSX)

S3 File The miRNA-mRNA interaction network. (XLSX)

S4 File The circRNA-miRNA network. (XLSX)

S5 File Identification of a subset of genes that showed concordant upregulation or downregulation across both T-cell populations. (XLSX)

S1 Fig Quality-control assessment of the scRNA-seq dataset before and after filtering. Violin plots show the distributions of three key quality metrics, including the number of detected RNA molecules per cell (*nCount_RNA*), the number of detected genes per cell (*nFeature_RNA*), and the percentage of mitochondrial transcripts (*mito.percent*), before quality control (upper row) and after quality control (lower row). The post-filtering data show reduced extreme values and improved overall data quality for downstream single-cell analysis.
(TIF)

S2 Fig Distribution of normalized expression values across samples. Box-and-whisker plots show the normalized expression distribution for each sample, including Healthy1, Healthy2, Healthy3, Patient1, Patient2, and Patient3, after data normalization. The comparable expression distributions across samples indicate that the normalization procedure reduced technical variation and improved data comparability for downstream analysis.
(TIF)

S3 Fig UMAP visualization of sample distribution before and after batch-effect correction. The left panel shows the single-cell transcriptomic data colored by sample identity before batch-effect correction, illustrating inter-sample variability across the dataset. The right panel shows the same data after batch-effect correction/harmonization, demonstrating improved mixing of cells from different samples and reduced technical variation across groups. Samples are labeled as HD1: Healthy Donor1, HD2: Healthy Donor2, HD3: Healthy Donor3, PD1: Patient Donor1, PD2: Patient Donor2, and PD3: Patient Donor3.
(TIF)
